# Life History and Production of the Western Gray Whale’s Prey, *Ampelisca eschrichtii* Krøyer, 1842 (Amphipoda, Ampeliscidae)

**DOI:** 10.1371/journal.pone.0147304

**Published:** 2016-01-22

**Authors:** Natalia L. Demchenko, John W. Chapman, Valentina B. Durkina, Valeriy I. Fadeev

**Affiliations:** 1 Laboratory of the Ecology of Shelf Communities, A.V. Zhirmunsky Institute of Marine Biology of the Far Eastern Branch of the Russian Academy of Sciences, Vladivostok, Russia; 2 Department of Fisheries and Wildlife, Hatfield Marine Science Center, Oregon State University, 2030 Marine Science Dr., Newport, Oregon, United States of America; 3 Laboratory of Cytophysiology, A.V. Zhirmunsky Institute of Marine Biology of the Far Eastern Branch of the Russian Academy of Sciences, Vladivostok, Russia; Virginia Commonwealth University, UNITED STATES

## Abstract

*Ampelisca eschrichtii* are among the most important prey of the Western North Pacific gray whales, *Eschrichtius robustus*. The largest and densest known populations of this amphipod occur in the gray whale’s Offshore feeding area on the Northeastern Sakhalin Island Shelf. The remote location, ice cover and stormy weather at the Offshore area have prevented winter sampling. The incomplete annual sampling has confounded efforts to resolve life history and production of *A*. *eschrichtii*. Expanded comparisons of population size structure and individual reproductive development between late spring and early fall over six sampling years between 2002 and 2013 however, reveal that *A*. *eschrichtii* are gonochoristic, iteroparous, mature at body lengths greater than 15 mm and have a two-year life span. The low frequencies of brooding females, the lack of early stage juveniles, the lack of individual or population growth or biomass increases over late spring and summer, all indicate that growth and reproduction occur primarily in winter, when sampling does not occur. Distinct juvenile and adult size cohorts additionally indicate growth and juvenile production occurs in winter through spring under ice cover. Winter growth thus requires that winter detritus or primary production are critical food sources for these ampeliscid populations and yet, the Offshore area and the Eastern Sakhalin Shelf ampeliscid communities may be the most abundant and productive amphipod population in the world. These *A*. *eschrichtii* populations are unlikely to be limited by western gray whale predation. Whether benthic community structure can limit access and foraging success of western gray whales is unclear.

## Introduction

The limits and production of ampeliscid amphipod populations are major concerns for whale conservation because they are critical prey of eastern and western North Pacific gray whales, *Eschrichtius robustus* (Lilljeborg, 1861) [1–5]. Recovery of the western gray whales (WGW from here on), since the ending of commercial whaling in the 1970s, has been slow relative to eastern gray whales (EGW). Eastern gray whales are no longer listed as threatened or endangered while the WGWs remain in the Red Book list [6]. We examined the life history and production of *Ampelisca eschrichtii* Krøyer, 1842, a critical prey species of WGWs in their Offshore feeding area on the northeastern Sakhalin Shelf of the Okhotsk Sea [7] to partially test whether WGWs could be food limited.

*Ampelisca macrocephala* Liljeborg, 1852 and other less abundant ampeliscid species are the primary prey of the eastern gray whales in their main feeding area, the Chirikov Basin of the Bering Sea [8]. Previous to the 1980s, Chirikov Basin ampeliscid populations were limited primarily by the epi-benthic flow of organic matter, intra- and inter-specific competition for space and reproductive success [9]. However, EGW numbers doubled between 1967 and the late 1980s [10–12]. The estimated 26,916 EGWs of 1987/1988 declined to 18,178 by the 1993/1994 census [11] and the EGWs have remained around 20,000 since 2002 [11–13]. Coyle et al. [8] estimated that the 2002/2003 EGWs consumed a significant fraction of *A*. *macrocephala* production in the Chirikov Basin and thus have limited ampeliscid populations since the late 1990s [14]. Punt and Wade [15] estimate that EGW are presently at around 85% of their prey carrying capacity. Coyle et al. [8] additionally proposed that the slow growth rates and the relatively long (4–5 year) life span of *A*. *macrocephala* could require decades for their populations to recover to densities observed previous to the 1980s. Climate-change related affects in the Bering Sea are also proposed as mechanisms that could limit gray whale food sources [16–18].

A potential for limited WGW food has similarly emerged as a particular concern in the western North Pacific. Weller et al. [19] identified 69 WGWs by 1998. The overall estimated WGW number remains at less than 200 to date and the annual increase in their numbers has declined each year since 2001 [13,20–23].

**Fig 1:**
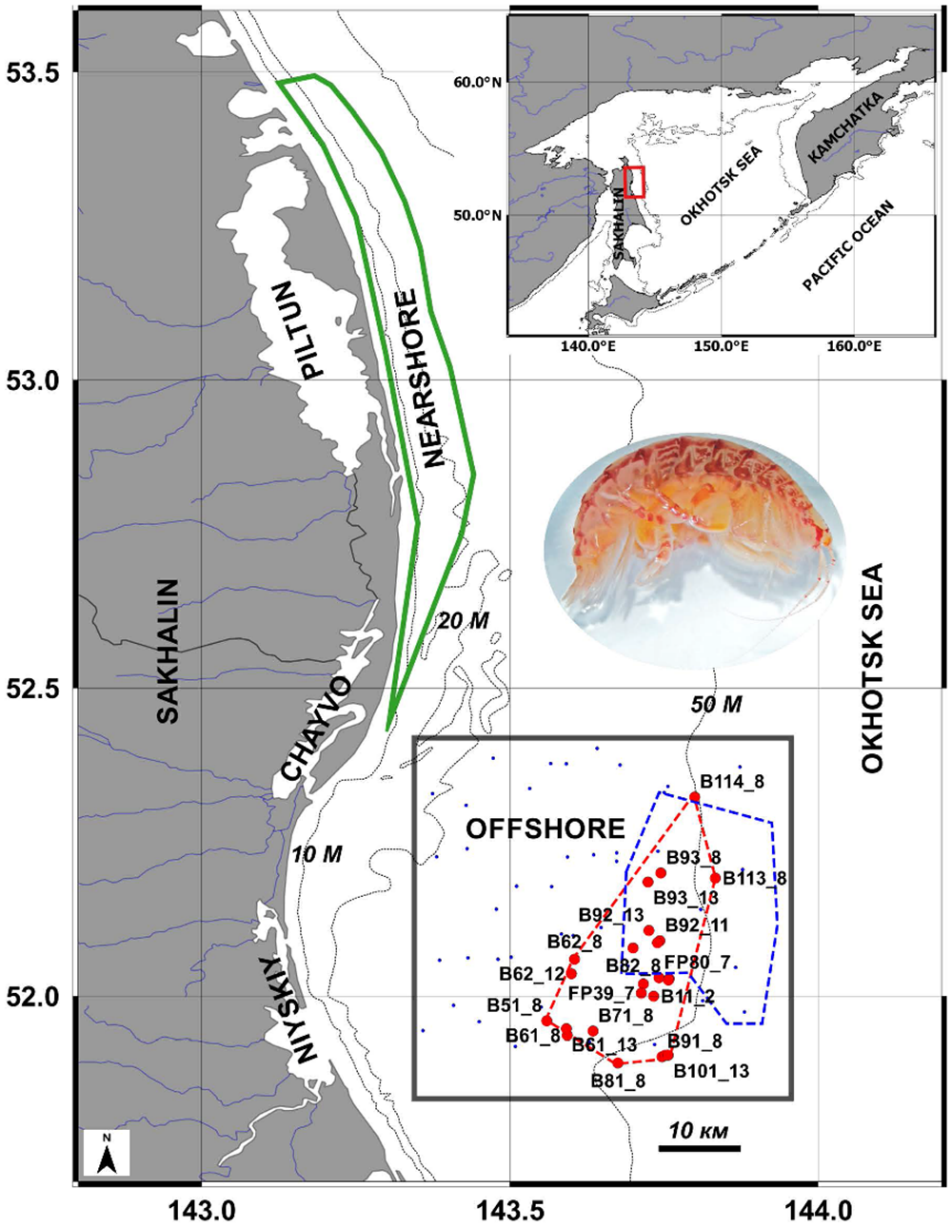
The Offshore feeding area. NE Sakhalin Island (solid red rectangle), 2011 western gray whale foraging perimeter (blue dashed line), Complex I-III sample sites (small blue dots), 2002–2013 Complex IV *Ampelisca eschrichtii* (photograph) area (red dashed line) including station designations followed by their year of sampling.

Ninety-seven of the 122 known western gray whales foraging on the Northeastern Sakhalin Shelf of the Okhotsk Sea in 2008 were sighted exclusively within the Nearshore area adjacent to the Piltun and Chayvo Lagoons (Fig 1) [20,24]. More than 40% of these whales occurred in the Piltun area, adjacent to Piltun Lagoon (52.7–53.4°N and 143.1–143.4°E) at less than 20 m depths and within 5 km of shore during the 2011 survey season [25]. Also in 2011, 37 to 83 whales were observed in the Offshore feeding area (Fig 1) [25] (approximately, 51.8–52.4°N and 143.4–143.9°E, southeast of Chayvo Bay, northeast of Niyskiy Bay and 30–45 km off of the Sakhalin coast in 40–60 m depths) [3,4,7,24,26–29]. The dimensions of these foraging areas are defined by the distributions of feeding whales observed primarily during aerial surveys [27]. The benthic communities of these areas are dominated by dense aggregations of gammaridean amphipods [30–32]. The restricted general position of the entire Offshore feeding area has been apparent in all sampling efforts since 2002 [7].

The Nearshore and Offshore feeding areas are within the region of highest primary and secondary production in the Okhotsk Sea [31,33–40]. Strong winds, tidal mixing, upwelling, and Amur River discharges over the northeastern Sakhalin Shelf induce intense diatom blooms in these areas during the ice free May-November period [37,41–43].

Fadeev [4,7] used cluster analysis of the macrobenthos abundance in the Offshore feeding area to identify four benthic complexes dominated by: sand dollars (Complex I), cumaceans and non-ampeliscid amphipods (Complex II), ampeliscid amphipods mixed with bivalves and sea anemones (Complex III) and densely dominant ampeliscids (Complex IV). Complex IV consists mainly of *Ampelisca eschrichtii* populations and covers the largest eastern and southeastern part of the study area (Fig 1 herein and in [7]). The Offshore area is assumed to be critically important for WGW feeding [7,24].

Ampeliscids construct mucous cemented sac-like tubes in dense aggregations on muddy, medium and fine sands in areas dominated by strong bottom currents [7,44–46]. Ampeliscids feed from their tubes on epibenthic particles including “phyto-detritus” [8] that they capture from the epibenthos and sediment surface with their second antennae [44,46]. Stable isotope ratios and fatty acid compositions of *A*. *eschrichtii* of the Sakhalin Shelf indicate that planktonic diatoms are the most important and ultimate *A*. *eschrichtii* food [47]. High Arctic shallow benthic food webs nevertheless, do not all vary directly with seasonal changes in primary production [48] and many benthic species rely on detrital pathways rather than direct primary production [49] which can dampen the impacts of large seasonal fluctuations in primary production.

In the Offshore Complex IV area, *Ampelisca eschrichtii* comprise >95% of the amphipod biomass and can exceed densities of 16,000 m^−2^ and reach biomasses of up to 1 kg m^−2^ [4,7,24,28]. Amphipods brood their young until they are released as fully formed juveniles and thus lack specialized larval dispersal stages. Quantitative benthic grab sampling is therefore sufficient for measuring entire amphipod populations. As in other high Arctic systems however, (e.g., *A*. *macrocephala* in the Chirikov Basin [50] and in Svalbard [51]), access to the Sakhalin shelf benthic communities has been possible only within the late June to early November ice free period [4,7,24,28]. The lack of access to these populations in winter months has confounded efforts to measure annual *A*. *eschrichtii* growth and production or to resolve the relations between *A*. *eschrichtii* production, primary production and detrital food pathways. The year to year contributions of these amphipods to the energy requirements of their WGWs predators has therefore also remained unclear. Expanded comparisons of all data from six years of sampling during summer months between 2002 and 2013, nevertheless, has permitted estimates of *A*. *eschrichtii* life history, population dynamics, production and biomass. These new results also allow comparisons of WGW prey abundance and production with EGW prey abundance and production.

Special permits were not required for the benthic invertebrate samples collected for this research. All information on WGW for this research is from previously published data. No interactions with endangered or threatened species occurred in the course of this research.

## Materials and Methods

We measured the population dynamics and production of *A*. *eschrichtii* from 45 samples collected at 24 stations in 44–60 m depths within the Sakhalin Offshore area between June and October in six sampling years spanning 2002 to 2013 (Fig 1 and S1 Table). We compared male/female ratios, the occurrence and frequencies of female reproductive conditions, the presence of terminal phase breeding males, the presence of newly hatched juveniles and the relative weights of similar sized reproductive males and females. We expected high proportions of brooding females, major changes in the frequencies of brooding females between early and late summer months and significant changes in size frequencies among different sampling months in our samples if production and growth occurs primarily in summer.

Bottom water temperatures on stations ranged between -0.8 and 9.2°C, salinities ranged between 30.8 and 32.3 psu, opacity (in Formazin Turbidity Units [FTUs]) ranged between 1.0 and 8.9 and oxygen saturation levels ranged between 83% and 90%. Sediments analyzed from 14 of the 24 stations and ranged between medium, fine and silty sands (S1 and S2 Tables). Sampling was completed within a few days of each year except in 2007, when sampling extended over a 75 day period between July and October (Table 1 and S1 Table).

**Table 1:** *Ampelisca eschrichtii* density, biomass and production. Day, month and year (**Date**), juvenile, adult and total density (respectively, **J** *m*^*-2*^, **A** *m*^*-2*^ and **N** *m*^*-2*^), juvenile, adult and total grams biomass (respectively, **J** *g m*^*-2*^, **A** *g m*^*-2*^ and **B** *g m*^*-2*^), grams production *m*^*-2*^ per sample (**P**_**s**_), production per biomass per sample (**P/B**_**s**_) estimated from juvenile and adult cohorts within sample dates (**P**_**s**_ and **P/B**_**s**_) and production per biomass estimated from juvenile and adult cohorts among consecutive years (**P**_**yr**_ and **P/B**_**yr**_) of the Offshore *A*. *eschrichtii* populations between 2002 and 2013 and their mean values from the normalized averages among the 6 survey years (see also S1 Table).

Date	J *m*^*-2*^	A *m*^*-2*^	N *m*^*-2*^	J *g m*^*-2*^	A *g m*^*-2*^	B *g m*^*-2*^	P_s_ *g m*^*-2*^	P/B_s_	P_yr_ *g m*^*-2*^	P/B_yr_
28/6/02	3,233	2,752	5,985	64.9	514.4	579.3	499.2	0.9		
23/7/07	10,950	2,400	13,350	245.5	460.3	705.8	1130.6	1.6		
17/8/07	6,640	2,560	9,200	140.6	378.0	518.6	581.8	1.1		
12/9/07	4,793	1,867	6,660	102.9	298.3	401.2	460.4	1.1		
5/10/07	3,600	2,010	5,610	93.3	284.7	378.0	324.7	0.9		
29/9/08	3,935	1,635	5,570	90.6	228.7	319.3	325.3	1.0	477.5	1.2
20/8/11	308	833	1140	9.6	137.1	146.7	76.0	0.5		
Av. Aug. 2012	2,684	1,060	3,744	25.7	207.5	233.2	348.5	1.5	112.4	1.0
16–19 Oct. 2013	1,485	1,175	2,660	27.1	222.9	250.0	228.1	0.9	347.6	1.4
Mean	3,023	1,611	4,634	60.6	277.6	338.2	350.3	1.0	312.5	1.2

Samples were collected using an 0.25 m^2^ Okean grab from the R/V *Bukhoro* in the 2002 TINRO-Center expedition and using an 0.2 m^2^ van Veen grab in the IMB expeditions from the R/V *Igor Maksimov* (in 2011) and R/V *Akademik Oparin* in all other years. These samples were washed through a series of sieves ending at 1.0 mm mesh size in 2002 and a mesh size of 0.5 mm in all following years. The retained organisms were fixed in 4% formalin and later, sorted out, identified, weighted, measured and then transferred to 75% ethanol. We compared density and biomass of *A*. *eschrichtii* among the 45 samples (S1 Table) with sediment composition at the 14 stations where sediment data were available (S1 and S2 Tables).

Amphipods from 20 samples and 17 of the 24 stations were selected randomly for morphometric analyses (S1 Table). Quantitative subsamples of *A*. *eschrichtii* from each of the major samples were taken to reduce processing efforts. We noted sex and reproductive development in 17 samples from 17 sites (S1 Table and Table 2).

**Table 2:** Reproductive development among months and years. Female reproductive stages (***F0-FIV***) and female/male ratio (**F/M**) of the Offshore *A*. *eschrichtii*.

Development	Jun-02	Sept-07	Sept-08	Aug-11	Aug-12	Oct-13	Mean
***F0***	92%	79%	87%	72%	73%	79%	80%
***FI***	3%	0%	13%	2%	11%	0%	5%
***FII***	0%	13%	0%	8%	0%	15%	6%
***FIII***	0%	0%	0%	0%	0%	0%	0%
***FIV***	5%	8%	0%	18%	16%	6%	9%
***F/M***	1.5	1.9	2.5	1.1	2.3	1.0	1.7

All *A*. *eschrichtii* in each subsample were weighed together to the nearest 5 mg and then subsets of these samples were counted, sexed, measured, blotted dry on filter paper and individually weighed to the nearest milligram. Body lengths were measured to the nearest 0.1 mm from the tip of the rostrum to the basis of the telson of outstretched *A*. *eschrichtii* using a calibrated ocular micrometer under a stereomicroscope. Males were distinguished by the presence of penile papillae and secondarily, by their relatively longer antennae, powerful pleopods and transversely pleated coxal gills. Females were distinguished by the presence of oöstegites and secondarily, by unpleated coxal gills.

We verified morphological classifications of juvenile, male and female reproductive development by histological analyses of 5 juveniles, 14 morphological males, 10 morphological females and a possible intersex specimen collected in October, 2013 (Table 3).

**Table 3:** Reproductive development by sex and length. Sex (assessed by morphology), estimated cohorts (C3-C11, see below), body length (mm), number of specimens (**N**), and reproductive development (assessed from morphology and histology of juveniles, males, females and an intersex specimen) of *A*. *eschrichtii* collected in October 2013.

Row	Morphological sex	Estimated cohort	Length (mm)	N	Gonad and reproductive development
1	juvenile	C3	8.8–9	5	no gonads
2	male	C5	13.5–14	2	no gonads
3	male	C6	16.5–17	2	no gonads
4	male	C7	18–19	2	spermatocytes
5	male	C7	20	2	spermatocytes, spermatids
6	male	C7	21.0–21.5	2	spermatocytes, spermatids
7	male	C9	24	2	spermatozoa, few spermatocytes and spermatids
8	male	C9	26	2	spermatozoa, few spermatocytes and spermatids
9	female (no brood)	C5	15	2	previtellogenic oocytes
10	female (no brood)	C8	22–24	2	vitellogenic oocytes, few previtellogenic oocytes
11	female (brooding embryos)	C9	23–25	2	resorption of vitellogenic oocytes, few previtellogenic oocytes (1 specimen); empty ovary (1 specimen)
12	female (empty brood pouch)	C11	32.5–33	2	previtellogenous oocytes
13	female (no brood)	C11	32.5–33	2	resorption of few vitellogenic oocytes, few previtellogenic oocytes (1 specimen); vitellogenic oocytes, few previtellogenic oocytes (1 specimen)
14	male (morphological intersex)	C10	30	1	spermatozoa

Each specimen used for histological analyses was soaked in fresh water for 24 h, dehydrated, cleared in xylene and then infiltrated with melted paraffin. The paraffin was cooled into blocks that were cut into 10 μm thick sections for mounting on slides for microscopy. Sections containing gonad tissue were stained using hematoxylin and eosin and permanently mounted on glass slides. The histology slides and whole dissected specimens for these analyses are deposited in the museum collections of the A.V. Zhirmunsky Institute of Marine Biology of the Far Eastern Branch of the Russian Academy of Sciences.

We assessed the sequence of reproductive development with size and with time (Tables 2 and 3) from the variation in reproductive development among male and female lengths and collection years. We followed Tzvetkova classification [52] of female reproductive condition:

*F0* –rudimentary oöstegites lacking egg retention setae;
*FI*–brooding uncleaved eggs;
*FII*–brooding cleaved eggs;
*FIII*–brooding fully formed juveniles;
*FIV*–fully developed oöstegites bearing egg retention setae and empty brood pouch.

We counted eggs, where egg loss during handling was not apparent.

We estimated *A*. *eschrichtii* weight increase per length using the power function:

$$W=a\times L^{b}$$ (1)

where, *W* is individual wet weight (g), *L* is body length (mm) and *a* and *b* are estimated coefficients. Weights of all specimens were estimated by this model where weight was not recorded. We compared deviations from exponential weight increase with length to assess potential growth limits and assumed that *Ampelisca* less than the estimated weight were underweight and *Ampelisca* greater than the estimated weight were above normal weight. We also used this relation to estimate total weight among length classes and biomass (*B*) of overall populations.

We identified sequential modes among 1 mm length-frequencies by the Bhattacharya and Normsep procedures in the FiSAT II software package [53,54]. The FiSAT II procedure distinguishes size modes by a separation index (S.I.) of greater than 2. We assumed that common FiSAT II identified length cohorts correspond to relative cohort ages and interpreted increasingly larger size frequency modes as sequentially older cohorts. We estimated changes in intra-annual growth, survival and biomass of the 2007 *A*. *eschrichtii* populations sampled in a 75 day period (July-October, S1 Table) from the changes in their length frequencies, densities and biomasses over time. We distinguished variations of population size structures among samples and years by cluster analysis (S1 Table, S2 Table and S1 Fig) in PRIMER v.6 statistical software [55]. We normalized these absolute frequencies by subtracting the mean and taking their square roots and constructing a similarity matrix (rows–length classes, columns–samples) based on the Bray Curtis coefficient for this analysis [56].

We estimated average annual *A*. *eschrichtii* production (*P*) over the *n* sampling years (*n* = 6) by cohort summation [57,58] where:

$$P=\frac{1}{n}\sum_{i=1}^{n}\left(\frac{\left(N_{1,i}+N_{2,i}\right)}{2}\times \left(W_{2,i}-W_{1,i}\right)\right)$$ (2)

in which, *N*_1,*i*_ is the number of 0+ juveniles m^-2^ in year *i*, *N*_2,*i*_ is the number of 1+ (one year older than the juveniles), adults m^-2^ in year *i*, *W*_1,*i*_ is the average wet weight of a juvenile, and *W*_2,*i*_ is the average weight of an adult in year *i*. We estimated production to biomass *P*/*B* from the average annual production divided by the average summer biomass m^-2^, *B*. We estimated production from the juvenile cohort of the first year relative to the adult cohort of the following year within sample years (2002, 2007, 2008, 2011, 2012 and 2013), among consecutive sampling years (2007–2008, 2011–2012 and 2012–2013) and by the normalized means of the six sample years (Table 1).

Sampling entirely within WGW feeding areas, defined by vessel based sightings, each year, is not possible and year to year positions of maximum *Ampelisca* beds also cannot be predicted. We therefore estimated the Offshore area overlap of 2007–2013 *A*. *eschrichtii* Complex IV sampling sites and WGW foraging (Fig 1) by the field perimeters 2011 WGW sightings in 2011 [25]. We estimated the overlapping areas of these perimeters by polygon triangles summation using their geographical coordinates [59]. We produced all maps using open source software QGIS v. 2.12.1-Lyon (http://qgis.org/ru/site/).

## Results

### Density and biomass

*A*. *eschrichtii* aggregations were in moderately and well sorted fine and medium sands with silt/clay content less than 25% (S1 Table, S2 Table and S1 Fig). We did not find a correlation between *A*. *eschrichtii* length frequencies and grain structure among samples (S1 Fig). However, *A*. *eschrichtii* abundances were positively correlated with percentage of medium sand (***r***^***2***^ = 0.5, ***df*** = 12, S1 and S2 Tables) and median grain size (***r***^***2***^ = 0.4, ***df*** = 12) and negatively correlated with percentage of fine sand (***r***^***2***^ = 0.5, ***df*** = 12). *A*. *eschrichtii* abundances were also weakly correlated with other species of *Ampelisca* among samples (***r***^***2***^ = 0.4, ***df*** = 12). The maximum abundance of *A*. *eschrichtii* among our samples was 13,350 spec. m^-2^ and maximum biomass was 705.8 g m^-2^ (July 2007, S1 Table).

### Reproductive development

**Fig 2:**
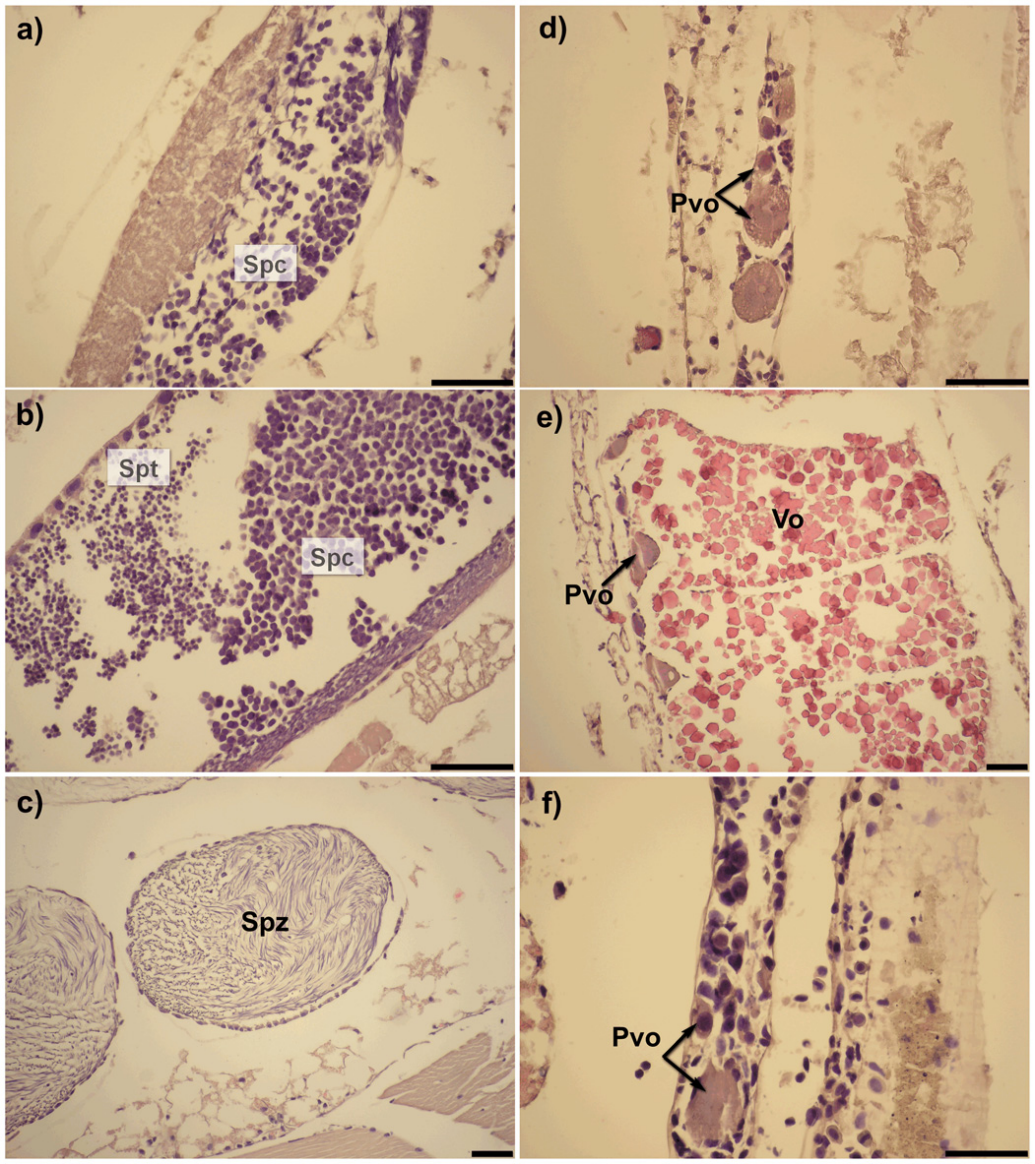
Histology of *A*. *eschrichtii* males and female gonads. **a)** –18 mm male testis; **b)**– 21 mm male testis; **c)**– 26 mm male testis; **d)**–ovary of 15 mm female at stage *F0*; **e)**– 22 mm female at stage *F0* and; **f)**– 32 mm female at stage *FIV*; Spc–spermatocytes, Spt–spermatids, Spz–spermatozoa in spermatophore, Pvo–previtellogenic oocytes, Vo–vitellogenic oocytes (scale = 100 μm).

Less than 12 mm length *A*. *eschrichtii* lack gonads and do not have suitable external morphologies for distinguishing sexes. We therefore classified all less than 12 mm length individuals as juveniles. Nearly all 12 to 17 mm length *A*. *eschrichtii* bear penile papillae that we initially misinterpreted as male characters. These individuals however, lack gonads (Table 3, rows 1–3) and thus cannot be functional males. Eight morphologically identified males greater than 18 mm in length (Table 3, rows 4–8) contained gonads that held spermatocytes (Fig 2a), spermatids (Fig 2b) and spermatozoa (Fig 2c). No males contained ova. Testes of the 18 mm male contained only spermatocytes (Table 3, Fig 2a) while the twenty mm length male testes contained spermatocytes and spermatids (Fig 2b). Spermatozoa were most abundant in the testes of 24 and 26 mm length males (Fig 2c). The advancing reproductive condition with size among the eight 18 mm and larger males indicates that only the largest males are reproductive (Table 3).

Results of our histological analyses of female and male gonads are consistent with a gonochoristic life history. The ovaries of 15 mm *F0* females contained previtellogenous oocytes (Fig 2d). The 22–24 mm *F0* female ovaries contained mostly vitellogenic oocytes that were completely formed and ready for oviposition and some previtellogenic oocytes (Fig 2e). The ovaries of 23–25 mm *FII* females (brooding cleaved eggs) were either empty or contained possibly resorptive vitellogenic oocytes and relatively few previtellgenous oocytes (Table 3, row 11). The large 32–33 mm *F0* females ovaries were filled either by previtellogenic oocytes (Table 3, Fig 2f) or by vitellogenic oocytes with a new generation of the previtellogenic oocytes (Table 3, row 13) which indicates that *A*. *eschrichtii* can produce at least 2 broods. Two morphological intersexes, 19 mm and 30 mm in length, among 428 *A*. *eschrichtii* specimens examined from the 18 October, 2013 samples, bore small oöstegites and male genital papillae. However, the 30 mm specimen lacked ovaries and its testes contained only spermatozoa (Table 3, row 14) and thus it did not appear to be an intersex or to have changed sex.

**Fig 3:**
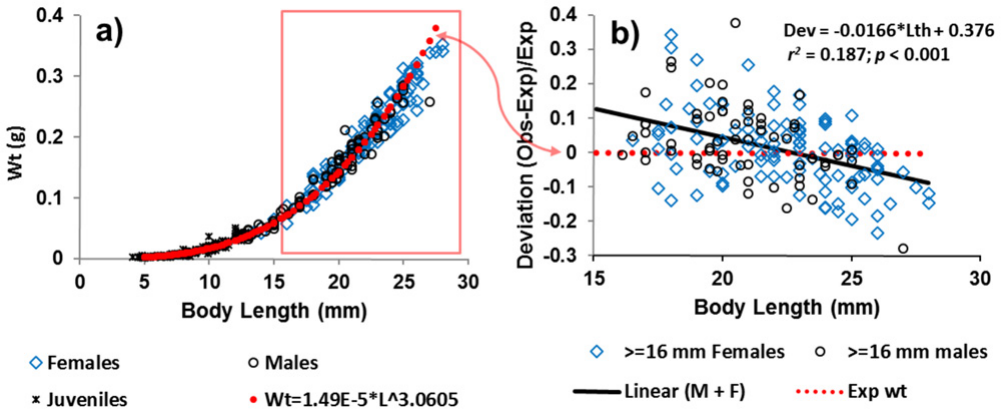
*Ampelisca eschrichtii* weight deviation with length. **a)** Estimated (red dashed line) and observed female, male, and juvenile wet weights by length (blue diamonds, black circles and orange crosses, respectively) (*r*^2^ = 0.99, *df* = 398, *p* < 0.001) collected in 2002, 2007, 2012 and 2013 with the red box enclosing greater than or equal to 16 mm males and females that; **b)** deviate from the expected weight (red dashes, Fig 3a and 3b) with increasing length (black line, *r*^2^ = 0.187, *df* = 167, *p* < 0.001).

Variations in length to weight among males and females also did not indicate sex change (Fig 3). The ranges of *A*. *eschrichtii* male and female lengths are similar among samples (Student’s test, ***p*** < 0.05) and the largest *A*. *eschrichtii* female by length (33 mm) in our samples, was collected at the same location 18 October, 2013 as the largest male (27 mm) that we found.

**Fig 4:**
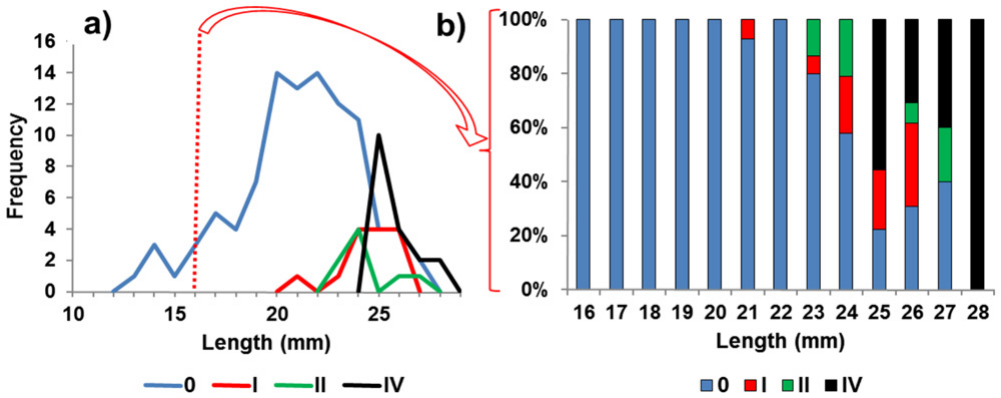
Female reproductive stages among length frequencies. **a)** 10–28 mm body length classes of stages *F0*, *FI*, *FII* and *FIV* from 2002, 2007, 2011, 2012 and 2013 combined with a vertical red line marking the greater than 16 mm size ranges included in; **b)** the percentages of reproductive stages *F0*, *FI*, *FII* and *FIV* among 16 mm and greater length females.

The smallest *F0* female confirmed by histology was 12 mm in length and bore small, bare oöstegites. *F0* females (with oöstegites lacking setae and lacking developing ova) ranged between 12 and 33 mm and occurred in all samples. We found 21 to 27 mm length *FI* females brooding uncleaved eggs in all August and September samples (Fig 4) and *FII* females ranging between 23 and 28 mm in length, brooding cleaved eggs (Fig 4) in the latest seasonal collections, mainly in mid-October, 2013 (Table 1).

Reproductive development of females is thus size dependent. All brooding females were greater than 20 mm in length and *FIII* females bearing juveniles were absent (Fig 4). The average female reproductive stages *F0*—*FIV* among all years were, respectively, 80%, 5%, 6%, 0% and 9% (Table 2). Thus, the combined average of stage *FI* and *FII* females among all months and years sampled was only 11%. Moreover, 20% of all females from all samples and years were of reproductive stages *FI* or greater (Table 2). Females brooding juveniles (stage *FIII*) were absent and all *FIV* females, that had released juveniles, were greater than 24 mm in length (Fig 4). The low frequency of egg bearing females (*FI* and *FII*) (Table 2) is not consistent with prolific reproduction during the June to October months sampled during this survey (S1 Table).

The ratio of morphological *A*. *eschrichtii* male per female among samples was 1:1.6 (Males = 1.02*Females– 6.3; ***r***^2^ = 0.73, ***df*** = 15, ***p*** < 0.01, Table 2). The low male frequencies can result from greater male mortality or to undercounting of small males. We did not find terminal-molt *A*. *eschrichtii* males in our samples, similar to those reported by Highsmith and Coyle [50] among *A*. *macrocephala* in the Chirikov Basin or by Mills [44] among *A*. *abdita* Mills, 1964 on the northwestern Atlantic shelf. Such males are expected during maximum reproductive periods. Increasing frequencies of lower than expected weights of large males and females among all sample months and years (Fig 3b) and the lack of terminal phase males are inconsistent with rapid growth or ongoing summer reproduction in these populations.

### Fecundity

The low frequency of egg bearing females in these summer/fall samples place egg count based estimates of annual fecundity in doubt. However, reproductive development in the *A*. *eschrichtii* females begins at approximately 15 mm with previtellogenic oocytes. All females with eggs or embryos were greater than 20 mm in length (Fig 4). Therefore, following the pattern of size related reproductive development, egg production and deposition into the brood pouch is completed when females reach 22–24 mm lengths (Fig 2d and 2e). Ovaries of the 23 and 25 mm *FII* females examined histologically, contained resorptive or vitellogenic oocytes and a few previtellogenic oocytes (Table 3) or they were empty. We found ova that we were unable to count in a dissected 25.5 mm female that was brooding 47 eggs. We also found 64 mature oocytes in a 24 mm female and 84 mature oocytes in 25 mm female. These females are likely to have brooded fewer eggs previously than the sum all ova available in their ovaries and thus, they were likely to have been iteroparous. The coincidence of ova in brooding and stage *FIV* females, the high ratios of ova to eggs and the broad size range of *FIV* females all indicate that *A*. *eschrichtii* are iteroparous.

### Length frequencies

**Fig 5:**
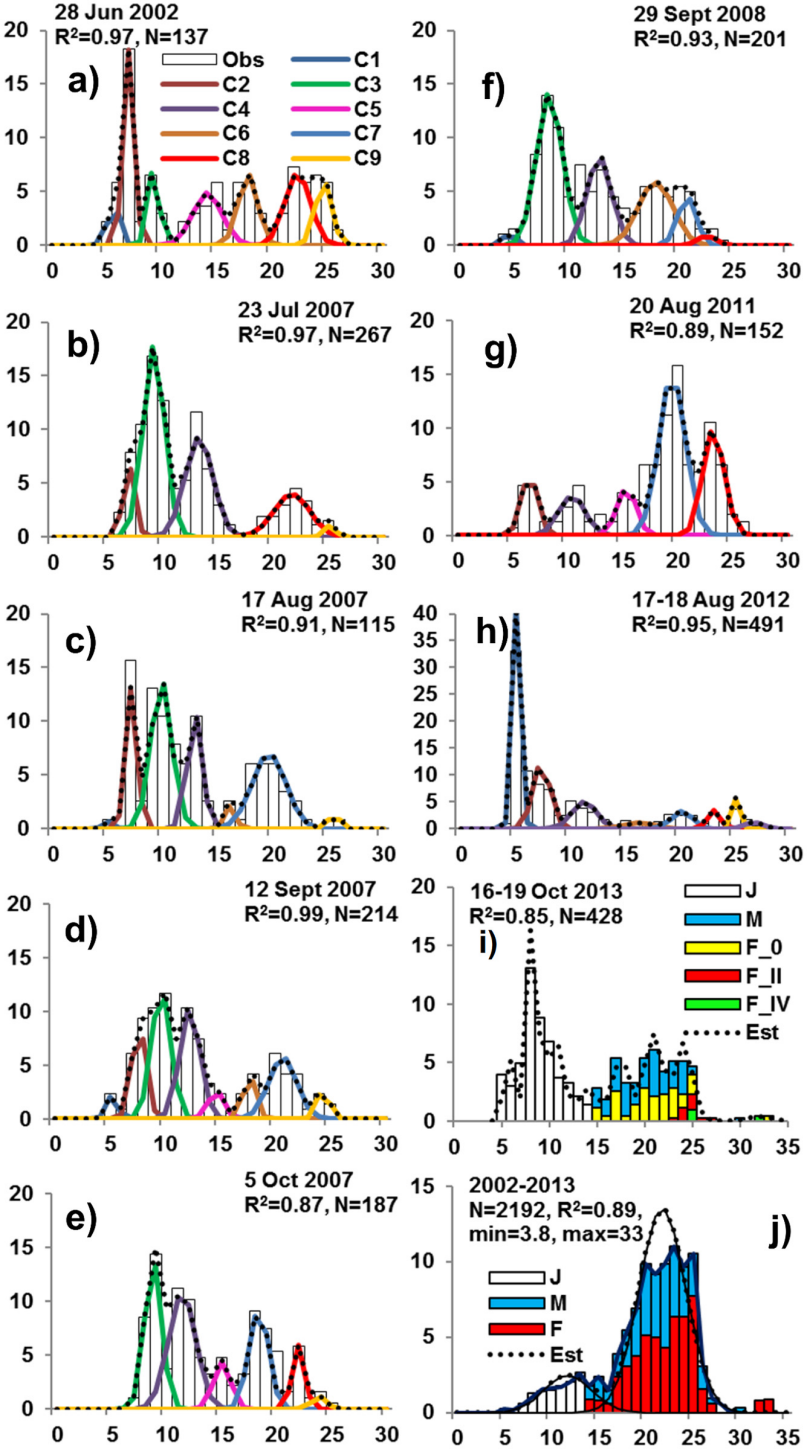
Relative cohort densities or biomass among years and months. Including (C1-C9) densities m^-2^ among 1 mm length frequencies of the 2002–2013 sampling dates (**a-i**) and the proportion of average biomass m^-2^ for all years (**j**) with number of specimens (N), observed frequencies (vertical bars), predicted frequencies (dashed lines), regressions of predicted and observed frequencies and example of juvenile and adult reproductive development and sex ratios in (**i**).

Six to eight minor length class modes were apparent in all samples and years (Fig 5a–5j) that converged into two major length class modes. The two major modes separated within the 15 to 17 mm length intervals were apparent in all yeas (Fig 5a–5j). The juvenile-adult division is particularly apparent when all years are averaged in proportions of weight (Fig 5j). The small size mode consisted of juveniles and the larger size mode consisted of mature males and females (Fig 5j). The 16 mm division is consistent only with length classes being separate age classes in contrast to instars. The smallest *A*. *eschrichtii* in our samples (3.8 mm in length) was collected 29 September, 2008. The low frequencies of such newly hatched juvenile length classes additionally indicate that our summer-fall sample populations (Fig 5a–5i) were not actively reproducing. The constant sex ratio we found over the sampling season was also inconsistent with active reproductive activities and high male mortality observed among other ampeliscids [46,60].

The proportions of distinguishable juvenile and adult size modes varied greatly among samples and years (Fig 5a–5i). A greater than 30 mm length cohort occurred only in 2013 (Fig 5i). All other cohorts in all other years were less than 28 mm in length (Fig 5a–5h). The low variation in length frequencies within 2007 samples (Fig 5b–5e) and greater size variation within samples from other years (Fig 5a and 5f–5i) indicates that variations in population structure occur mainly among years rather than among sample locations. A low frequency of 15 to 17 mm length classes divides juveniles from reproductive males and females, in all samples (Fig 5a–5j). The 15–17 mm division is also consistent with reproductively mature length classes being one year older than the juveniles. Alternative potential growth and development, including longer ampeliscid life spans (> 4 years) [45], would obscure this separation.

**Fig 6:**
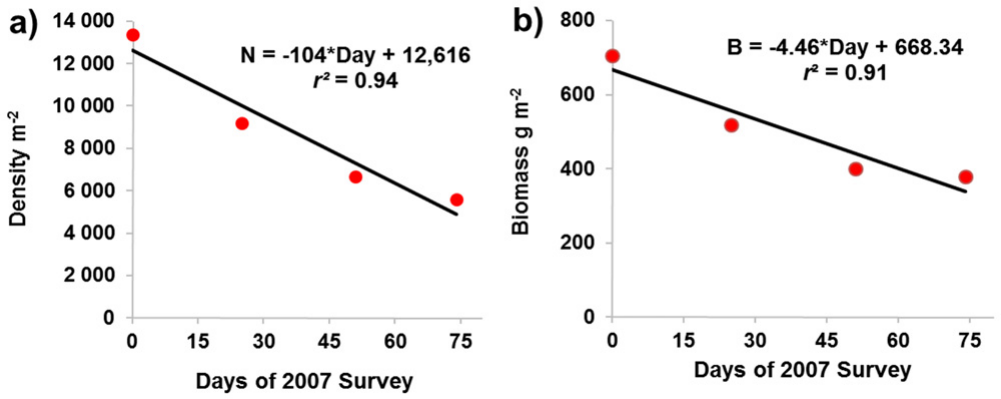
Summer declines of *Ampelisca eschrichtii*. ***a***) density and **b)** biomass over the 75 day sampling period (23 July-5 October, 2007).

The six to eight minor length frequency modes among the July-October samples of 2007 (Fig 5b–5e) were apparent also in other years (Fig 5a and 5f–5i) and are also likely to consist of individual cohorts. Average lengths of the individual cohorts and of the composite juvenile and adult cohorts over the 75 day sampling period of 2007 did not vary significantly (Fig 5b–5e) and thus, individual growth was not apparent in the 2007 sampling period. Moreover, the densities and biomasses of the 2007 the *A*. *eschrichtii* populations declined (Fig 6).

The two major length modes we found in all years indicate that the lack growth among the 2007 cohorts was not due to random sample variation or to exceptional growth conditions in 2007. The bimodal population structure, separating juveniles and reproductive adults therefore is a likely result of a two-year *A*. *eschrichtii* life span and juvenile release outside of summer months. We therefore assumed a two-year cycle for our estimates of annual production.

### Production

We estimated production based on the 0+ year juvenile (≤16 mm length) and 1+ year adults (> 16 mm length) cohorts identified above (Fig 5) among samples (Table 1, ***P***_***s***_), from 0+ year juveniles of previous years relative to 1+ adults in consecutive following years (Table 1, ***P***_***yr***_) and from the averages of average year estimates (Table 1, **Means**). Although our estimates of biomass, production and energetics (Tables 1 and 4) include conservative parameters for the Offshore feeding area relative to the Chirikov Basin *A*. *macrocephala* and to other ampeliscids [8], the energetics of the two feeding areas appear dramatically different (Table 4).

**Table 4:** Gray whale and ampeliscid energetics. In the Offshore (Okhotsk Sea) and Chirikov Basin (Bering Sea) feeding areas.

Gray Whale–Ampeliscid Energetics	Offshore	Chirikov	Source
Estimated gray whales area^-1^	83	4,760	**1**
Area km^2^ (*A*. *eschrichtii* biomass >300 g m^-2^)	372	8,900	**2**
km^2^ gray whale^-1^	4.5	1.9	
Mean Ampeliscid biomass (g m^-2^ wet wt)	338.2	125.6	**3**
Mean Ampeliscid biomass (t wet wt area^-1^)	125,821	1,117,840	
Ampeliscid production per biomass yr^-1^ (P/B)	1.0	0.9	**4**
Standing wet Ampeliscid biomass (t Whale^-1^)	1,516	235	
Ampeliscid production (t wet wt area^-1^)	119,947	1,006,056	
Ampeliscid production (t wet wt Whale^-1^)	1,445	211	
Ampeliscid biomass (g dry wt m^-2^)	127	62.4	**5**
kCal g^-1^ dry wt *Ampelisca* spp.	4.1	4.1	**6**
GW Kcal requirements yr^-1^	1.6x10^8^	1.6x10^8^	**7**
Total Kcal production yr^-1^	1.8x10^11^	2.0x10^12^	
Sustainable gray whales (15% of production)	173	1,921	**8**

Sources: **1)** [8,20,23,29]; **2)** herein and [8]; **3)** *A*. *eschrichtii*, 2002–2013 (Table 1 herein), *Ampelisca* spp.–[8] (Table 4, 1973–74, 1986–88, 2002–03); **4)** *A*. *eschrichtii*, 2002–2013 (Table 1 herein), *Ampelisca* spp. [8]; **5)** *A*. *eschrichtii* herein, 2002–2013, *Ampelisca* spp. [8] (Table 3, 2002–03, and Table 4 conversion of wet to dry wt 0.375); **6)** [8] (Table 3, 1973–74, 1986–88, 2002–03); **7)** [8,50]; **8)** [8] (Accepting previous assumptions that consumption of less than 15% of average annual ampeliscid production is sustainable).

Thirty-nine percent of the 564 km^2^ 2011 WGWs foraging ground in the Offshore was overlapped by the 665 km^2^ 2002–2013 ampeliscid sampling area (Fig 1). *A*. *eschrichtii* occurred in 95% of all samples in the benthic “Complex IV” of the Offshore [28]. However, sea urchins *Echinarachnius parma* (Lamarck, 1816) present in 17% of samples from the whole Offshore feeding area and the predominance of gravel in 17% of all samples there [28] were likely to limit the value of these sites and locations for WGW foraging. Thus, up to 34% of the Offshore area could be unsuited for WGWs foraging. We therefore estimate, conservatively again, that the effective WGWs foraging ground of 2011 was about 372 km^2^ (Table 4).

The Offshore WGW feeding area (that includes ampeliscid biomasses >300 g m^-2^) is about 4% of the EGW Chirikov feeding area (Table 4). The maximum density of WGWs observed in the Offshore between 2002 and 2013 was less than half as great as EGW densities in the Chirikov area and the *A*. *eschrichtii* biomass per WGW in the Offshore area was 6 times the ampeliscid biomass per EGW in the Chirikov feeding area (Table 4). WGWs also had 8 times the ampeliscid production per whale than was available to EGWs in the Chirikov Basin (Table 4). Thus, ampeliscid biomass and production is greater in WGW feeding areas than in EGW feeding areas. These data nevertheless, do not challenge previous conclusions of food limitation for EGW [8,10,14].

## Discussion

Northeastern Sakhalin Shelf *Ampelisca eschrichtii* have a predominantly two-year gonochoristic, iteroparous life cycle in which growth occurs primarily under ice cover. These populations therefore must require detritus or other winter based primary production food sources. Moreover, these abundant *A*. *eschrichtii* populations are unlikely to be limited by WGW predation.

Although the proportions of *A*. *eschrichtii* individuals within particular length modes varied dramatically among years and samples, adjacent length modes invariably clustered, with 3–4 juvenile length classes and 3–4 adult length classes divided by a low frequency gap at around 16 mm. We infer that the 16 mm division was overgrown by the adult cohorts of our summer samples in the previous winter but not reached by the juvenile cohorts produced in the previous winter. The 3–4 reproductively adult size classes in our summer populations were thus most likely to have been new juveniles two winters previously. Asynchronous and unequal growth rates over increasingly diverse ages force individual length classes of long lived species to overlap and merge. The widely separated length modes of *A*. *eschrichtii* are consistent only with a relatively short (2 year) life span and synchronous (winter-spring) growth among size classes.

Our observations of underweight reproductive sized males and females, a lack of newly hatched (less than 4 mm length) juveniles, the absence of *FIII* brooding females and the absence of secondary phase reproductive males are consistent with a lack of summer growth. The declines of abundances and biomasses of the Offshore *A*. *eschrichtii* between early summer and late autumn of 2007 are also consistent with winter or early spring reproduction and biomass production outside of our sample periods.

The increasing sizes of consecutive female reproductive stages identified by histology are consistent with no more than two broods in any winter and 3 broods (rarely) over a life span. Stage *FI* and *FII* females that we found are likely to be the first to reproduce in winter. Stage *F0* females, having greater than average weights per length, are likely to produce the second winter cohort of juveniles. The largest and underweight stage *FIV* females of summer are likely to produce the third or fourth winter/spring juvenile cohorts. Survival of reproductive sized *A*. *eschrichtii* over two summers that we possibly observed in the 10^th^ (30 mm) cohort of 2013, appears to be rare. Thus, nearly all individuals in our samples were less than two years old. *A*. *eschrichtii* is thus gonochoristic and reproduces once or, rarely, twice during each extended winter and spring period of ice cover.

Our inferred life history of *A*. *eschrichtii* is consistent with previous observations of high latitude ampeliscid biology. Fifteen of the 18 benthic amphipod species on the west Spitsbergen coast, including *A*. *eschrichtii*, incubate eggs during the polar night and release their offspring in early April [51]. Broods of *A*. *eschrichtii* collected in summer could have been deposited before sampling commenced. The lack of early stage juveniles in the 5 month sampling period also indicates that the eggs we found were not hatching over the sampling period. These observations are consistent with the life history of *A*. *macrocephala* in the Øresund in which breeding occurs after October and is followed by the complete absence of mature males in later months [46]. The absence of less than 5 mm *A*. *macrocephala* juveniles in the Chirikov Basin [9] similarly indicates that these populations also carry developing eggs over summer and fall that hatch in winter. Our estimates of ***P*/*B*** are within the range of previous estimates for ampeliscids [60].

Although diatoms must be the ultimate food source of *A*. *eschrichtii* [47], winter growth of *A*. *eschrichtii* on the northeastern Sakhalin Shelf occurs after most diatom production has ceased and settled to the benthos as phytodetritus [61]. Large populations of *A*. *eschrichtii* occur throughout the Okhotsk Sea, including the western Kamchatka Shelf [62], the eastern Sakhalin Shelf [30,31] and Sakhalinskiy Bay [63] of the northernmost Sakhalin Island. Pasternak [63] proposed that *A*. *eschrichtii* aggregations in fine and silty sands, adjacent to silty muds at the mouth of the Sakhalinskiy Bay form in response to optimal currents and detritus accumulation. A winter dependence on detrital food is common among other high latitude benthic amphipods [48,49]. WGWs that forage on *A*. *eschrichtii* are therefore likely to depend on detrital pathways from primary production of previous summers.

The production and biomass of the Offshore area *A*. *eschrichtii* ([7,28] and herein) exceeds the previous record for ampeliscids [64] and may be the highest for any amphipod population in the world [60]. More than 99% of the summer *A*. *eschrichtii* biomass is among individuals that are greater than the critical 8 mm in length required to be retained by WGW baleen [2,65]. The Offshore area is not the most extensive WGW feeding area in Far Eastern Russia [20] and alternative WGW feeding areas remain in use each year. If WGW forage optimally, their continuous use of all feeding areas (including Offshore) indicates that they are of similar value. Our estimated high WGW food stocks correspond with previous observations [7,24]. The high production rates of the Offshore area ampeliscid populations provide significant potential to quickly recover from intense predation. Since other feeding areas are likely to contain similar prey sources as Offshore, the limited growth of the WGW feeding aggregate, numbering less than 200, off northeastern Sakhalin [20] is unexpected when 1,500–10,000 WGW are estimated to have been in the region in the 19th century [66].

All regions of the North Pacific are likely to be accessible to gray whales [13]. However, massive immigration by EGWs to the seemingly abundant Western Pacific food sources or unusually rapid growth of the WGW numbers has not occurred. Unless Allee effects [67] or mortality sources, such as predation, unsanctioned whaling, diseases and parasites are greater among WGWs than among EGWs, the potential mechanisms for food limitation of all Pacific gray whales warrants reconsideration. Prey abundance is necessary but not sufficient to preclude food limitation. Not all sites in the Offshore area where the highest *Ampelisca* biomasses occur appear to be used by WGW (Fig 1). Ineffective, naïve or otherwise inefficient foraging behaviors resulting from genetic or social bottlenecks [13] or prey refuges could limit WGW access to these prey [28]. *Ampelisca* populations in coarse sediments, for instance, could be less accessible to foraging whales [28]. Intense fishing could also alter benthic community composition on the Sakhalin Shelf and expand the distributions of inedible species that reduce WGW access to benthic prey [28]. Climate changes may also limit WGW foraging by altering primary production, timing and extent of ice cover, detritus distribution and the consequent benthic communities and sediments. More information on the conditions of benthic communities that affect WGW foraging success and winter sampling to resolve the winter life history and ecology of these amphipods would greatly benefit WGW conservation efforts.

## Supporting Information

S1 Fig Sediment associated *A*. *eschrichtii* length frequencies. Clusters AI, AII and B (left) of 2002 to 2013 correspond to the decreasing frequencies of large and older *A*. *eschrichtii* (right) among sediment types: Sf–fine sand; Sls–silty sand; Sm–medium sand and; Ssl–sandy silt (data in S1 and S2 Tables).
(PDF)

S1 Table Sample and population data. Sampling dates, site designations, replicates per station, latitude (N) and longitude (E), depth, bottom temperature (T), practical salinity units (PSU), sediment type, number of measured specimens (n, ind), individuals m^-2^ (N m^-2^) and estimated grams biomass m^-2^ (B, g m^-2^).
(PDF)

S2 Table Granulometry. Sites containing *Ampelisca eschrichtii* in 2007 and 2008 with predominant grain size highlighted in red.
(PDF)
