# Supplementary material for: Life History and Production of the Western Gray Whale’s Prey, Ampelisca eschrichtii Krøyer, 1842 (Amphipoda, Ampeliscidae)
Source: PLoS One. 2016 Jan 22;11(1):e0147304. doi: 10.1371/journal.pone.0147304 (PMC4723087; doi:10.1371/journal.pone.0147304)
Supplement: S1 Fig — Clusters AI, AII and B (left) of 2002 to 2013 correspond to the decreasing frequencies of large and older A. eschrichtii (right) among sediment types: Sf–fine sand; Sls–silty sand; Sm–medium sand and; Ssl–sandy silt (data in S1 and S2 Tables). (PDF) [file pone.0147304.s001.pdf]

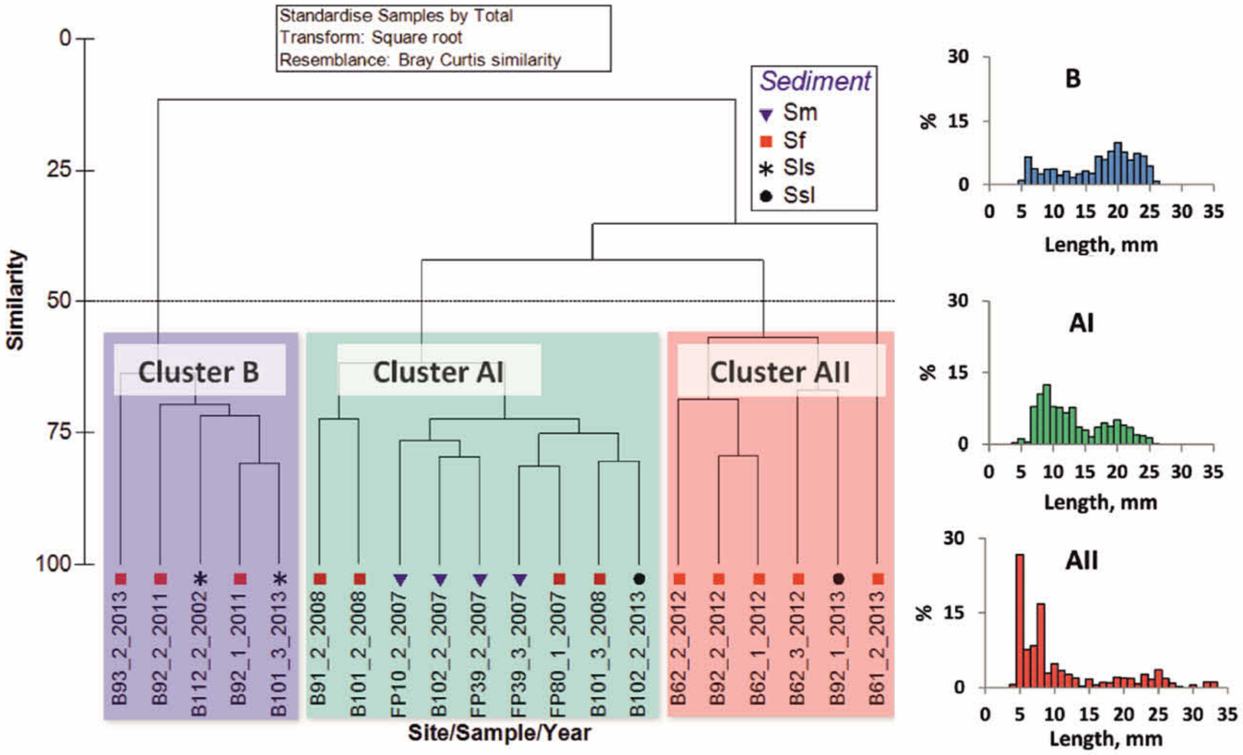

S1 Fig. Sediment associated *A. eschrichtii* length frequencies. Clusters AI, AII and B (left) of 2002 to 2013 correspond to the decreasing frequencies of large and older *A. eschrichtii* (right) among sediment types: Sf – fine sand; Sls – silty sand; Sm – medium sand and; Ssl – sandy silt (from S1 and S2 Tables).
