# Supplementary material for: Life History and Production of the Western Gray Whale’s Prey, Ampelisca eschrichtii Krøyer, 1842 (Amphipoda, Ampeliscidae)
Source: PLoS One. 2016 Jan 22;11(1):e0147304. doi: 10.1371/journal.pone.0147304 (PMC4723087; doi:10.1371/journal.pone.0147304)
Supplement: S1 Table — Sampling dates, site designations, replicates per station, latitude (N) and longitude (E), depth, bottom temperature (T), practical salinity units (PSU), sediment type, number of measured specimens (n, ind), individuals m-2 (N m-2) and estimated grams biomass m-2 (B, g m-2). (PDF) [file pone.0147304.s002.pdf]

S1 Table. Sample and population data. Sampling dates, site designations, replicates per station, latitude (N) and longitude (E), depth, bottom temperature (T), practical salinity units (PSU), sediment type, number of measured specimens (n, ind), individuals m^{-2} (N m^{-2}) and estimated grams biomass m^{-2} (B, g m^{-2}).

Date	Site_Yr	Repli-cates	Longitude	Latitude	Depth, m	T, °C	PSU	Sediment	n, ind.	N m^{-2}	B, g m^{-2}
28.06.2002	B11_2[a]	2	143.7333	52.0000	52	-0.8	–	Sls	137	5,985	579.3
23.07.2007	B102_7	2	143.7583	52.0283	54	2.2	32.3	Sm	267	13,350	705.8
17.08.2007	FP10_7[b]	2	143.7167	52.0200	52	4.8	32.1	Sm	115	9,200	518.6
12.09.2007	FP39_7[b]	2, 3	143.7133	52.0050	53	6.1	31.8	Sm	214	6,660	359.9
05.10.2007	FP80_7[b]	1	143.7420	52.0300	53	9.0	30.9	Sf	187	5,610	378.0
09.09.2008	B82_8	1-3	143.7000	52.0783	50	5.4	32.0	Sf	–	3,013	364.2
09.09.2008	B114_8	1-3	143.8000	52.3233	53	4.6	32.2	Sf	–	643	134.4
29.09.2008	B51_8	1-3	143.5600	51.9600	44	9.0	31.2	Sf	–	203	28.3
29.09.2008	B61_8	1, 2	143.5933	51.9367	47	8.8	31.3	Sf	–	155	19.7
29.09.2008	B62_8	1-3	143.6050	52.0600	45	9.2	31.1	Sf	–	667	72.9
29.09.2008	B71_8	1-3	143.6350	51.9435	51	8.4	31.5	PbGr	–	1,270	124.2
29.09.2008	B81_8	1-3	143.6753	51.8922	57	8.9	31.3	Sf	–	9,067	485.9
29.09.2008	B91_8	2	143.7468	51.9023	60	8.4	31.6	Sf	40	6,400	237.9
29.09.2008	B93_8	1-3	143.7450	52.2000	49	8.8	31.3	Sf	–	2,320	78.1
29.09.2008	B101_8	2, 3	143.7518	51.9048	60	8.5	31.5	Sf	161	4,740	274.9
29.09.2008	B113_8	1, 2	143.8333	52.1917	56	8.5	31.3	Sf	–	1,200	53.9
20.08.2011	B92_11	1, 2	143.7433	52.0900	51	–	–	Sf	152	1,140	146.7
17.08.2012	B92_12	2	143.7395	52.0868	52	3.9	32.1	Sf	69	2,760	212.5
18.08.2012	B62_12	1-3	143.6000	52.0365	44	–	–	Sf	422	5,627	266
16.10.2013	B93_13	2	143.7241	52.1854	48	3.8	31.6	Sf	42	1,680	221.1
17.10.2013	B92_13	1	143.7256	52.1067	50	–	–	Ssl	54	2,160	62.7
18.10.2013	B61_13	2	143.5918	51.9473	46	4.8	30.8	Sf	32	1,280	172.2
18.10.2013	B101_13	3	143.7568	51.9050	59	2.9	32.1	Sls	109	4,360	444.1
19.10.2013	B102_13	2	143.7573	52.0262	53	3.9	31.6	Ssl	191	3,820	354.6

Note. PbGr – pebble and gravel, Sf – fine sand; Sls – silty sand, Sm – medium sand, Ssl – sandy silt, [a] – material of L.L. Budnikova and R.G. Bezrukov (2003), [b] – feeding sites of gray whales.
