# Supplementary material for: Life History and Production of the Western Gray Whale’s Prey, Ampelisca eschrichtii Krøyer, 1842 (Amphipoda, Ampeliscidae)
Source: PLoS One. 2016 Jan 22;11(1):e0147304. doi: 10.1371/journal.pone.0147304 (PMC4723087; doi:10.1371/journal.pone.0147304)
Supplement: S2 Table — Sites containing Ampelisca eschrichtii in 2007 and 2008 with predominant grain size highlighted in red. (PDF) [file pone.0147304.s003.pdf]

**S2 Table. Granulometry.** Sites containing *Ampelisca eschrichtii* in 2007 and 2008 with predominant grain size highlighted in red.

| | | | | **Sedimentary fractions** | | | | | | |
|---|---|---|---|---|---|---|---|---|---|---|
| | | | | **PbGr** | **Sc** | **Sm** | **Sf** | **Al+Pe** | | |
| **Site** | **Md** | **So** | **Sk** | **>1.0** | **1-0.5** | **0.5-0.25** | **0.25-0.1** | **<0.1** | **Sediment** | **Sorting** |
| B102_7 | 0.34 | 0.82 | 1.0 | 2.0 | 4.4 | 74.8 | 6.3 | 12.5 | medium sand | MWS |
| FP10_7 | 0.28 | 0.65 | 0.7 | 0.9 | 1.5 | 55.1 | 30.8 | 11.7 | medium sand | MS |
| FP39_7 | 0.33 | 0.80 | 1.0 | 0.4 | 4.8 | 72.8 | 10.4 | 11.6 | medium sand | MS |
| FP80_7 | 0.17 | 0.79 | 1.0 | 0.0 | 0.0 | 11.1 | 88.9 | 0.0 | fine sand | MS |
| B101_8 | 0.15 | 0.75 | 1.0 | 0.1 | 1.2 | 5.8 | 73.6 | 19.3 | fine sand | MS |
| B91_8 | 0.14 | 0.74 | 0.9 | 0.3 | 0.1 | 2.6 | 73.0 | 24.0 | fine sand | MS |
| B51_8 | 0.15 | 0.79 | 1.0 | 0.1 | 0.8 | 2.9 | 88.7 | 7.5 | fine sand | MS |
| B61_8 | 0.16 | 0.80 | 1.0 | 0.2 | 1.4 | 3.3 | 89.7 | 5.4 | fine sand | MS |
| B62_8 | 0.15 | 0.81 | 1.0 | 0.0 | 0.0 | 0.8 | 96.5 | 2.7 | fine sand | MS |
| B81_8 | 0.15 | 0.76 | 1.0 | 1.3 | 1.8 | 2.9 | 75.0 | 19.0 | fine sand | MS |
| B82_8 | 0.15 | 0.74 | 1.0 | 0.0 | 0.0 | 14.5 | 66.9 | 18.6 | fine sand | MS |
| B93_8 | 0.16 | 0.81 | 1.0 | 0.1 | 0.5 | 1.8 | 95.9 | 1.7 | fine sand | MS |
| B113_8 | 0.14 | 0.78 | 1.0 | 0.0 | 0.0 | 1.6 | 81.8 | 16.6 | fine sand | MS |
| B114_8 | 0.15 | 0.76 | 1.0 | 1.7 | 3.2 | 6.0 | 75.4 | 13.7 | fine sand | MS |

Note. MWS – moderately well sorted, MS – moderately sorted, Md – median grain size, So – sorting coefficient, Sk – skewness.
